# Switch to faricimab after initial treatment with aflibercept in eyes with diabetic macular edema

**DOI:** 10.1007/s10792-024-03226-2

**Published:** 2024-06-25

**Authors:** Francesco Pichi, Abdulhamid Abdi, Shaikha Aljneibi, Ibraheem El Ghrably, Aniruddha Agarwal, Nicola G. Ghazi

**Affiliations:** 1 Eye Institute, Cleveland Clinic Abu Dhabi, PO Box 112412, Al Maryah Island, Abu Dhabi, United Arab Emirates; 2 Cleveland Clinic Lerner College of Medicine, Case Western Reserve University, Cleveland, OH USA; 3 Department of Physiology and Immunology, College of Medicine and Health Sciences, and Biotechnology Center, Khalifa University of Science and Technology, Abu Dhabi, United Arab Emirates; 4 Department of Ophthalmology, Maastricht University Medical Center+, Maastricht, The Netherlands

**Keywords:** Aflibercept, Diabetic macular edema, Faricimab, Switch

## Abstract

**Purpose:**

To assess the effectiveness of a switch to faricimab in individuals affected by DME and previously treated with aflibercept.

**Methods:**

In this retrospective, single-center study, DME patients previously treated with at least 3 injections of aflibercept then switched to faricimab were enrolled. Best corrected visual acuity (BCVA) and central subfield thickness (CST) were recorded at baseline, at the time of the switch and at 6 months follow-up. At transition to faricimab, patients were categorized as "good visual responders" (≥ 5 letters from baseline) or "poor visual responders" (< 5 letters), and as "good anatomical responders" (any reduction in edema compared to baseline) or "poor anatomical responders" (no reduction or worsening of edema). Changes in BCVA and CST were recorded at 6 months after the switch to faricimab.

**Results:**

100 eyes of 100 patients (61 female, 61%) were switched to faricimab after a mean of 6.8 ± 3.3 aflibercept injections. At the 6 months follow-up, only “poor visual responders” (N = 62) demonstrated a meaningful increase in BCVA (Δswitch-6M =  + 5 letters; *P* = 0.007), coupled with a reduction in CST (Δswitch-6M = − 67.9 µm; *P* = 0.004); participants with “poor anatomical response” upon transitioning exhibited a significant functional gain (Δswitch-6M =  + 4.5 letters; *p* = 0.05) but limited CST enhancements (Δswitch-6M = − 95.1 µm; *p* = 0.05).

**Conclusions:**

Faricimab shows a positive impact on anatomical and functional metrics in DME cases refractory to aflibercept.

## Introduction

Diabetic macular edema (DME) continues to significantly contribute to vision impairment and blindness worldwide in individuals who have diabetes. Presently, clinical trials with solid level 1 evidence have indicated that agents targeting antivascular endothelial growth factor (VEGF), namely ranibizumab and aflibercept, along with off-label bevacizumab, are the most efficacious therapeutic choices for enhancing visual acuity and macular structure in cases of center-involved DME when compared to laser treatment [1–3]. The trials RISE-RIDE for ranibizumab, VIVID-VISTA for aflibercept, and DRCR Retina Protocol T for bevacizumab have illustrated that nearly 40% of patients witnessed an improvement of 15 or more letters on Snellen eye charts after 2 years of follow-up [1–5].

Nevertheless, a subset of patients exhibit suboptimal responses to anti-VEGF therapy, and approximately 30% of DME patients continue to experience persistent DME even after a year of adequate treatment [6, 7]. Additionally, the number of injections administered in clinical practice is generally fewer than those in clinical trials, leading to suboptimal visual outcomes [9].

Therefore, it seems reasonable to consider transitioning between different anti-VEGF agents if the initial treatment fails to address macular edema sufficiently. Several small-scale studies have shown positive changes in both anatomical and functional aspects when DME patients switch from ranibizumab or bevacizumab to aflibercept [10–13], albeit with relatively short follow-up periods ranging from 1 to 6 months. On the contrary, other small studies suggest an improvement solely in anatomical aspects, with no significant alteration in visual function upon switching to aflibercept [14–17].

Recently, the emergence of faricimab, a bispecific antibody targeting both angiopoietin-2 (Ang-2) and VEGF-A, has revitalized prospects for enhanced treatment approaches in DME [18]. By concurrently inhibiting two distinct pathways involved in angiogenesis, faricimab offers the potential for a more robust and sustained response, thereby reducing treatment frequency and enhancing the quality of life for DME patients [19].

In theory, faricimab could exhibit enhanced efficacy in cases of DME that exhibit inadequate responsiveness to aflibercept, attributed to its capacity for Ang-2 inhibition [18]. Consequently, this study aimed to assess the effectiveness of a shift to faricimab in individuals affected by DME and previously treated with aflibercept.

## Materials and methods

This study is a retrospective single-center investigation. The data analyzed consisted of consecutive patients with diabetic macular edema (DME) who transitioned from receiving intravitreal injections of aflibercept to faricimab injections at the Eye Institute of Cleveland Clinic Abu Dhabi. The study period ranged from August 2022 to July 2023. The study was approved by the Ethics Committee of Cleveland Clinic Abu Dhabi, adhered to the principles outlined in the Declaration of Helsinki regarding research involving human subjects.

The inclusion criteria were as follows: (1) age of 18 years or older; (2) diagnosis of either type 1 or type 2 diabetes mellitus (DM); (3) clinically significant, center-involving DME according to EDTRS guidelines; (4) best-corrected visual acuity (BCVA) ranging from 20/200 to 20/20; (5) central subfield thickness (CST) equal to or greater than 300 μm, as measured by spectral domain optical coherence tomography (SD-OCT); (6) a minimum of 3 previous aflibercept intravitreal injections; (7) a minimum follow-up period of 6 months after the anti-VEGF switch.

The reason for switching was an effort to reduce the number of intravitreal injections.

The exclusion criteria were: (1) macular edema caused by factors other than diabetic retinopathy (e.g., retinal vein occlusion, age-related macular degeneration, postsurgical macular edema); (2) vitreo-retinal interface disorder such as vitreo-macular traction and epiretinal membrane; (3) significant media opacity that impeded the quality of OCT imaging (e.g., corneal opacity, cataract, vitreous hemorrhage); (4) history of ocular trauma or surgery within 6 months prior to the first faricimab injection; (5) prior administration of intravitreal corticosteroids before faricimab injection; (6) intravitreal anti-vascular endothelial growth factor (anti-VEGF) treatment (bevacizumab, ranibizumab, or aflibercept) within 1 month prior to the faricimab injection; (7) uncontrolled glaucoma, defined as intraocular pressure (IOP) exceeding 25 mm Hg despite the use of antiglaucoma medication in the study eye.

We collected demographic information (age and gender), hemoglobin A1c (HbA1c) levels, and the duration of DME for each patient. Best-corrected visual acuity was measured using standard ETDRS charts at three time points: at baseline diagnosis of DME prior to aflibercept intravitreal treatment, at the time when the patients switched from aflibercept intravitreal injections to faricimab injections, and at the 6 months follow-up post switch. At the time of the transition to faricimab, patients were categorized as either "good visual responders" (with an improvement of ≥ 5 letters from baseline) or "poor visual responders" (with an improvement of < 5 letters).

The two functional and the two anatomical groups were analyzed s in a post hoc analysis.

Throughout the study period, all patients underwent a comprehensive ophthalmic evaluation, which included slit-lamp biomicroscopy, applanation tonometry, fundus biomicroscopy, and SD-OCT (Spectralis HRA, Heidelberg Engineering, Heidelberg, Germany). The CST was measured at baseline, at the time of the switch to faricimab, and at the 6 months follow-up. Based on the CST measurements at the time of the switch, patients were classified as either "good anatomical responders" (showing any reduction in edema compared to baseline) or "poor anatomical responders" (showing no reduction or worsening of edema compared to baseline).

When patients were switched to faricimab, the decision was to treat using an as-needed regimen (which followed an OCT-guided treatment protocol).

The primary endpoint was the mean variation in BCVA between the pre-switch time and month 6 after the switch.

### Statistical analysis

Statistical analysis including descriptive statistics for demographics and main clinical records, and comparative analysis (Student’s *t*-test analysis for independent and paired samples and one-way analysis of Variance with Bonferroni correction) were performed through the open access R software (R Studio Version 1.1.383, R Project, www.r-project.org).

Continuous variables were reported as the median and interquartile range, while categorical variables were presented as frequencies and percentages. A *P* value of 0.05 or less was considered statistically significant. If a case had missing data for any of the variables, it was excluded from the analysis. Main outcome of the study was to evaluate if there is a significant clinical benefit of switching patients on existing anti-VEGF therapy to Faricimab.

## Results

Overall, 100 eyes of 100 patients (61 female, 61%), mean age 60 ± 9 years, were included in the analysis and followed for 8.4 ± 1.8 months after switching. Twelve patients were affected by type 1 DM (12%) and mean HbA1c was 8.4% ± 1.6%. Baseline clinical characteristics, stratified on the functional and anatomical outcome at anti-VEGF switch, did not vary among groups.

At switch to faricimab, data sub-analysis based on visual and anatomical response performed after a mean of 6.8 ± 3.3 aflibercept injections over a period of 11.3 ± 6.8 months, revealed that 62 eyes (62%) had a “poor visual response” (− 4.3 ± 10.7 letters), while 38 eyes (38%) disclosed “good visual response” (+ 13.9 ± 9.2 letters). Stratifying patients on the basis of OCT changes, 50 eyes (50%) showed no edema lowering or edema worsening, while 50 eyes (50%) showed any CST improvement. Patients with poor visual response were significantly associated with more limited morphological improvement (Fisher’s exact test for categorical variables *P* = 0.03).

Mean number of faricimab injections over the follow-up period of 6 months was 2.9 ± 0.9.

**Fig. 1:**
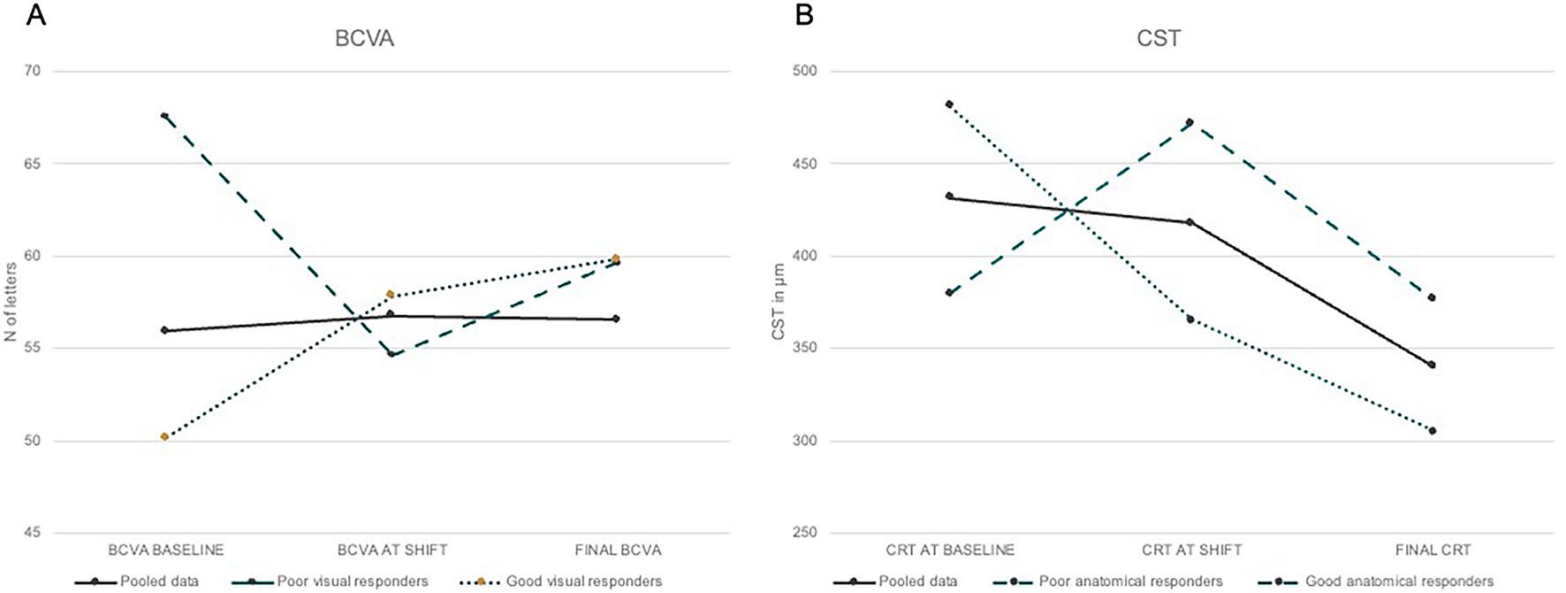
Pooled as well as individual line plots summarizing the changes in BCVA (**A**, functional response, *P* = 0.04) and CST (**B**, anatomical response, *P* < 0.001) in study subjects

Globally, within the study cohort, there was an observed but statistically non-significant decline in visual function (Δ_switch-6M_ = − 0.2; *P* = 0.04, Student’s *t*-test for paired samples; Fig. 1A) alongside a notable enhancement in anatomic structure on OCT (Δ_switch-6M_ =  − 77.4 μm; *P* < 0.001, Student’s *t*-test for paired samples; Fig. 1B) following a 6-month transition to faricimab.

When stratified by best-corrected visual acuity (BCVA) outcomes, only individuals with suboptimal visual responses demonstrated a meaningful increase in BCVA subsequent to the switch to faricimab injections (59.6 ± 18 letters, Δ_switch-6M_ =  + 5; *p* = 0.007, Student’s *t*-test for paired samples; Fig. 1A), coupled with a reduction in CST (331 ± µm, Δ_switch-6M_ = − 67.9 µm; *p* = 0.004, Student’s *t*-test for paired samples; Fig. 1B). Conversely, those initially exhibiting favorable visual responses to aflibercept showed only marginal BCVA changes in response to faricimab (59.8 ± 21 letters, Δ_switch-6M_ =  + 2; *p* = 0.2, Student’s *t*-test for paired samples; Fig. 1A), despite a substantial improvement in morphological parameters (345.1 ± µm, Δ_switch-12M_ = − 82 µm; *p* = 0.002, Student’s *t*-test for paired samples; Fig. 1B) (Table 1).

**Table 1:** Best corrected visual acuity (BCVA) and central subfield thickness (CST) at baseline, at the time of switch from intravitreal injections of aflibercept to faricimab, and at the 6 months follow up of faricimab treatment. Data are reported for the entire 100 eyes with diabetic macular edema, for "good visual responders" (with an improvement of ≥ 5 letters from baseline), "poor visual responders" (with an improvement of < 5 letters), "good anatomical responders" (showing any reduction in edema compared to baseline) and "poor anatomical responders" (showing no reduction or worsening of edema compared to baseline)

	BCVA change			CST change	
	Pooled patients (n = 100)	< 5 letters improvement (n = 62)	≥ 5 letters improvement (n = 38)	≤ 0 µm lowering (n = 50)	> 0 µm lowering (n = 50)
BCVA baseline, mean	55.9	67.5	50.2	56.5	56.2
BCVA at switch, mean	56.8	54.6	57.8	56.25	58.1
BCVA final, mean	56.6	59.6	59.8	60.75	59.8
CST baseline, mean	431.6	376.3	460.4	379.6	481.3
CST at shift, mean	417.7	398.9	427.1	471.9	365.6
CST final, mean	340.3	331	345.1	376.8	305.5

Analyzing the cohorts according to changes in CST, participants with no initial reduction or even an increase in CST upon transitioning exhibited a greater functional gain (60.75 ± 13 letters, Δ_switch-6M_ =  + 4.5; *p* = 0.05, Student’s *t*-test for paired samples;) in comparison to those manifesting any degree of edema reduction after aflibercept treatment (60.75 ± 9 letters, Δ_switch-6M_ =  + 1.7; *p* = 0.3, Student’s *t*-test for paired samples) (Fig. 1A). In terms of anatomical response (Fig. 1B), individuals with suboptimal anatomical improvements displayed limited CST enhancements with the use of faricimab (376.8 ± µm, _Δswitch-6M_ = − 95.1 µm; *p* = 0.05, Student’s *t*-test for paired samples), whereas those with any edema reduction after transitioning showed a more substantial CST improvement (305.5 ± 87 µm, Δ_switch-6M_ = − 59.1 µm; *p* = 0.07, Student’s *t*-test for paired samples) (Table 1).

## Discussion

In the present study we analyzed the functional and anatomical response in DME patients after switching from aflibercept to faricimab, and found that only patients with a poor visual and anatomical response to aflibercept benefit from the switch in terms of BCVA and CST. Patients who were responding to aflibercept either functionally or anatomically, do not exhibit a statistically significant increase in BCVA after the switch to faricimab.

Although the utilization of anti-VEGF injections has exhibited improvements in both the structural and functional outcomes for patients afflicted with DME, a subset of individuals display less-than-optimal responses [6, 7]. While the principal of switching remains controversial, when clinicians encounter such situations, they often face the decision of switching therapy to either intravitreal corticosteroids or attempting an alternative anti-VEGF agent. Given its comparably lower propensity for adverse effects, particularly among younger patients, the adoption of an alternative anti-VEGF agent is often deemed more favorable over corticosteroids.

A limited number of investigations have evaluated the results of transitioning to aflibercept following prolonged anti-VEGF treatment for persistent DME. Lim et al. [20] documented noteworthy enhancements in both functional and structural aspects after switching to aflibercept in 21 eyes from 19 DME patients who had demonstrated unsatisfactory responses to numerous bevacizumab/ranibizumab injections. The approach to aflibercept treatment post-transition varied in this study, with a median of 3 injections over an average 5-month follow-up and a 2.4-month interval between each aflibercept injection. Bahrami et al. [21] similarly showcased the favorable impact of aflibercept on visual and morphological improvements among DME patients with suboptimal responses to prior bevacizumab injections. Wood et al. [15], however, exclusively observed morphological enhancements with aflibercept among patients with inadequate responses to ranibizumab and/or bevacizumab injections, albeit the majority (11 of 14) were evaluated following only a single aflibercept injection. Rahimy et al. [22] similarly noted a mere morphological response to aflibercept injections subsequent to previous bevacizumab/ranibizumab therapy, attributing this outcome to irreversible functional damage stemming from prolonged DME.

Notably, the transition to aflibercept resulted in anatomical amelioration for the majority of patients across all these studies. Aflibercept possesses notably greater affinity for VEGF-A compared to bevacizumab or ranibizumab, and it additionally binds with VEGF-B and placental growth factor (PGF) [23]. The latter is a cytokine that can stimulate angiogenesis and plays a crucial role in the activation and maintenance of the inflammatory switch associated with neo-angiogenesis. Placental growth factor has been implicated in the pathogenesis of diabetic retinopathy and DME [24].

Based on previous findings, it has been observed that about 30% of patients exhibit DME resistant to effective treatment for a year [6, 7]. Even following 3 years of regular aflibercept treatment, 13% of DME patients continue to necessitate frequent dosing [25].

Faricimab, a bispecific antibody that simultaneously inhibits Vascular Endothelial Growth Factor (VEGF) and Angiopoietin 2 (ANG2), has been proposed as a novel approach to treat diabetic macular edema [19].

The role of VEGF in the pathogenesis of multiple blinding eye diseases has been well established in the literature. Despite its integral role in preserving the vascular equilibrium across various cellular structures and tissue types under normal physiological conditions, it has been implicated in the molecular etiology and pathogenesis of retinopathies associated with a range of ocular pathologies, including age-related macular degeneration (AMD), diabetic retinopathy (DR), as well as diabetic macular edema (DME). Therefore, therapeutic interventions that inhibit VEGF and its related pathways have been instrumental in averting visual impairment in a significant population of ocular disease patients, especially diabetic macular edema. Another potential therapeutic target is the cytokine ANG2, which is implicated in both angiogenesis and immune response modulation, thereby presenting as a promising candidate for the management of exudative AMD and other retinal pathologies. Observations of elevated intraocular ANG2 concentrations in patients with diabetic retinopathy and retinal vein occlusion underscore the potential clinical relevance of ocular ANG2 inhibition [19].

Faricimab, a novel bispecific antibody developed by Roche/Genentech, targets both VEGF-A and ANG2. It was approved by the FDA in 2022 for the treatment of wet AMD (w-AMD) and DME after meeting primary endpoints in phase III clinical trials, namely YOSEMITE and RHINE [19]. Both trials were randomized multi-center studies. In the YOSEMITE study, 73.8% of patients, and in the RHINE study, 71.1% of patients, experienced similar improvements in BCVA with faricimab compared to aflibercept. Notably, this was achieved with fewer injections [19, 28]. Furthermore, the anatomical response was also positive—reduction in intra-retinal fluid and central subfield thickness was more significant in the Faricimab arm of both trials. In line with earlier research, Faricimab demonstrated an overall good tolerability, and the occurrence of adverse events was comparable across the groups under study.

These findings suggest that faricimab may improve the visual and anatomical outcomes of patients with fewer injections than are necessary using the previous anti-VEGF injections.

In the present study, aflibercept-resistant DME patients who after a mean of 6.8 injections did not achieve a functional improvement (N = 62) benefited from a switch to faricimab injections both in terms of BCVA (+ 5 letters gained at 6 months, *P* = 0.007) and of CST (− 67.9 µm CST reduction at 6 months, *P* = 0.004). In addition, 50 patients who were not responding to aflibercept injections in terms of CST reduction, achieved a statistically significant functional result at 6 months after switching to faricimab (+ 4.5 letters gained at 6 months, *P* = 0.05).

The greater increase in BCVA and reduction in macular thickness in the non-responders patients who were switched might be explained by the blocking of all isoforms of VEGF along with blocking of ANG2. However, this improvement in visual acuity with faricimab may also be related to patients' inherent characteristics rather than features of faricimab. In addition to all these possible explanations, patients treated with repetitive aflibercept injections may demonstrate tachyphylaxis or a diminished therapeutic response to this agent over time as suggested in a great number of studies [26].

To our knowledge, very few previous study investigated the effectiveness of Faricimab in treating anti-VEGF-resistant DME [27]. Rush and Rush [28] investigated this aspect via a retrospective review of DME patients who had been receiving Aflibercept therapy. Subjects were divided into a study group which was switched to faricimab as well as a control group which continued with aflibercept. Their results revealed that a significant subset of the study group (37.5%) achieved a CST of less than 300 µm without retinal fluid on OCT after the 4-month study period, compared to 3.7% in the control group. Moreover, 41.7% of the study group experienced an improvement of two or more lines of visual acuity, in contrast to 11.1% in the control group. Both findings were statistically significant. The study concluded that faricimab can improve the short-term visual and anatomic outcomes in treatment-resistant DME patients formerly managed with aflibercept.

On the other hand, our results showed that DME patients who were responding to aflibercept injections either functionally (N = 38) or anatomically (N = 50), did not benefit from the switch to faricimab (respectively + 2 and + 1.7 letters gained at 6 months). This goes along with recent findings from Protocol T showing that selected patients with suboptimal anti-VEGF response at the 12-week mark exhibited improved BCVA at the 2-year point without altering their anti-VEGF agents, suggesting a delayed positive response in certain cases [29]. This may suggest that some DME cases are predominantly driven by the VEGF pathway such that regardless of the agent used, when VEGF blockade is achieved, a good clinical outcome results.

Our study's scope is confined by its retrospective design. The decisions regarding treatment and the frequency of visits or treatments were contingent upon the preferences of individual ophthalmologists, potentially introducing bias in the selection process for faricimab injections, and varying the number of previous aflibercept injections prior to the treatment shift, which ranged from 3 to 10.

Moreover, the absence of a control group for patient comparison leaves room for the possibility that sustained aflibercept treatment, regardless of initial response, could have led to gradual enhancements in both CST and BCVA over an extended period.

Further limitations are attributed to the relatively brief follow-up period, potentially impacting our ability to thoroughly assess the enduring effectiveness of faricimab. Notably, faricimab's introduction in the UAE in May 2022, with its prescription at Cleveland Clinic Abu Dhabi commencing in September 2022, resulted in a limited pool of eligible patients due to the short timeframe for faricimab administration. While the 6-month follow-up was not designed for long-term treatment assessment, it aimed to validate the efficacy of faricimab treatment in cases where other therapies for DME had proven unsuccessful.

Additionally, the duration of DME, treatment history, count of anti-VEGF injections, and intervals between visits and injections varied across participants, precluding a direct head-to-head comparison between faricimab's efficacy and that of prior anti-VEGF therapies.

Notwithstanding the inherent limitations of this study, we present real-world data with the longest follow-up period to date, suggesting a consistently positive impact on anatomical and functional outcomes when transitioning from aflibercept to faricimab for poorly responding DME cases. At the same time, patients with a good visual and anatomical response to aflibercept should continue treatment as there seems to be no benefit in switching anti-VEGF agent. However, larger scale studies with longer follow-up and a control arm are imperative to pinpoint the specific patient subgroup that may benefit from treatment switching, along with the optimal timing for switching.

## Data Availability

No datasets were generated or analysed during the current study.
